# NIR light-activatable dissolving microneedle system for melanoma ablation enabled by a combination of ROS-responsive chemotherapy and phototherapy

**DOI:** 10.1186/s12951-023-01815-4

**Published:** 2023-02-22

**Authors:** Fan Liu, Zeneng Cheng, Hanxi Yi

**Affiliations:** 1 Department of Neurology, Xiangya Hospital, Central South University, Changsha, China; 2 Division of Biopharmaceutics and Pharmacokinetics, Xiangya School of Pharmaceutical Sciences, Central South University, Changsha, China; 3 Department of Pathology, School of Basic Medical Science, Central South University, Tongzipo Road 172, Changsha, 410000 China; 4 Department of Pathology, Xiangya Hospital, Ultrapathology (Biomedical Electron Microscopy) Center, Central South University, Changsha, China

**Keywords:** Microneedles, Transdermal drug delivery, Liposomes, Reactive oxygen species, Prodrug, Synergistic therapy, Malignant melanoma

## Abstract

**Background:**

As a consequence of the aggressive and recurrent nature of melanoma, repeated, multimodal treatments are often necessary to cure the disease. While microneedle (MN)-based transdermal drug delivery methods can allow drugs to avoid first-pass metabolism and overcome the stratum corneum barrier, the main challenges of these delivery methods entail the lack of controlled drug release/activation and effective imaging methods to guide the entire treatment process.

**Methods:**

To enable a transdermal delivery method with controllable drug release/activation and effective imaging guidance, we designed a near-infrared (NIR) photoactivatable, dissolving MN system comprising dissolvable polyvinylpyrrolidone MNs arrays (MN-pB/I) containing liposomes that were co-loaded with the photosensitizer indocyanine green (ICG) and the reactive oxygen species (ROS)-activatable prodrug of doxorubicin (pB-DOX).

**Results:**

After applying the MN patch to the tumor site, the liposomes concentrated in the needle tips were released into the tumor tissue and distributed evenly upon dissolution of the matrix to enable targeted delivery. Then, the ROS produced by ICG after exposure to NIR light performed photodynamic therapy and activated the pB-DOX for chemotherapy by cleaving the prodrug moiety and converting it to DOX. As a dye, ICG was also used to guide the treatment regimens and monitor the efficacy by fluorescence and photoacoustic imaging. The growth of the tumors in the MN-pB/I group were inhibited by 93.5%, while those were only partially inhibited in the control groups. Negligible treatment-induced side effects and cardiotoxicity were observed.

**Conclusion:**

The MN-pB/I represents a multimodal, biocompatible theragnostic system with spatiotemporal control that was capable of ablating melanoma tumors after a single dose, providing a promising candidate for clinical melanoma therapy.

**Graphical Abstract:**

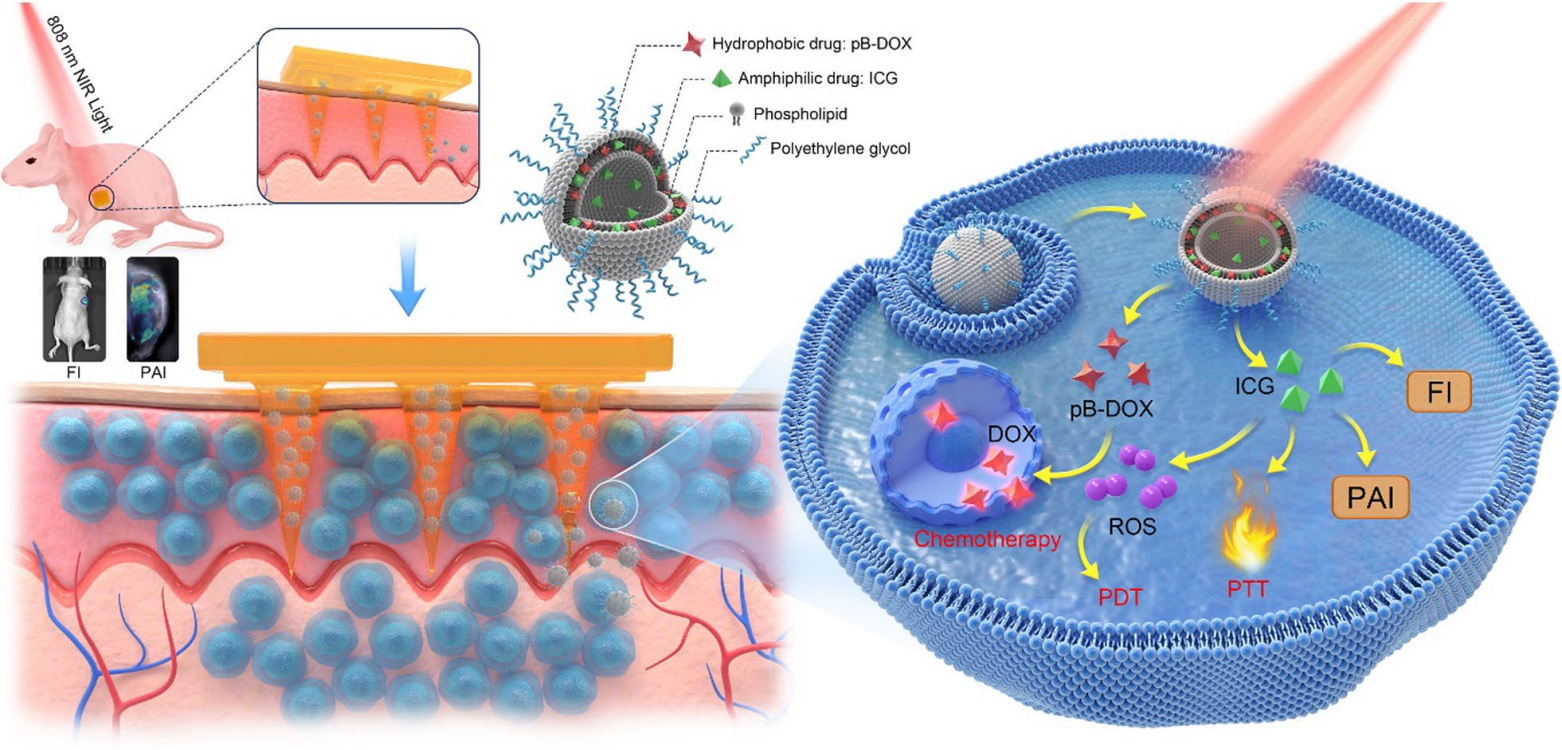

## Introduction

Melanoma is a common type of malignant skin cancer characterized by rapid progression and metastasis [1, 2]. Although malignant melanoma accounts for only 4% of all skin cancer cases, it is responsible for 79% of skin cancer mortalities [3, 4]. Currently, the main treatments for melanoma are surgery and chemotherapy [5]. However, surgical excision is not appropriate for patients with surgical contraindications or with melanoma lesions involving acra and mucosa that may be disabling, such as the eyes, mouth, nose, and vagina [6]. On the other hand, conventional chemotherapy manifests severe systemic toxicities because of the lack of targeted drug distribution [7, 8]. Therefore, at present, the benefit of surgery and chemotherapy often does not outweigh the risk, so there is an urgent demand to develop more advanced and effective melanoma treatment strategies.

Nanocarrier-based transdermal drug delivery systems (NTDDSs) have attracted significant attention as an alternative to chemotherapy for melanoma treatment not only because the drugs that are delivered can avoid first-pass metabolism, but also the local delivery of the drugs leads to significantly fewer side effects in normal tissues and organs involved in systemic circulation [9–11]. NTDDSs reported in the literature have been based on a variety of different vehicles, including liposomes, inorganic nanoparticles (NPs), solid lipid NPs, dendritic macromolecules, micelles, and nanogels; the integration of drugs into these vehicles can not only improve the physical and chemical stability of the drugs [12–15] but also provide a combination of intelligent delivery, multi-modal imaging, and combination therapy [16, 17]. However, NTDDSs suffer from poor skin penetration, which limits the efficacy of the drugs because malignant melanoma lesions extend below the dermal layer [18, 19]. Due to the complete non-invasive nature of nanocarriers [20, 21], the biggest challenge of NTDDSs is to overcome the stratum corneum (SC) barrier and improve the penetration depth and distribution of drugs [22–25].

Recently, microneedles (MNs) have been developed as a novel transdermal delivery method. MNs are composed of micron-length needle arrays that can penetrate the SC and form hundreds of micro-pathways, allowing the drugs loaded into the MNs to bypass the SC layer [26, 27]. MN-assisted DDSs provide a noninvasive (topical-transdermal) and invasive (injectable) drug administration approach to greatly the improve transdermal efficiency and tissue distribution of drugs compared to known NTDDSs as well as enable high patient compliance and the feasibility of self-administration [28, 29]. MNs are capable of delivering drugs directly to malignant melanoma lesions within the eyes, nose, nail, and vagina, where surgery is unsuitable, which offers great prospects for the treatment of acral and mucosal melanoma [6]. Nevertheless, MNs have limitations in not only the amount of drug that can be directly loaded but also the penetration depth and intratumoral distribution of the drug, since free drugs typically rely on passive diffusion [30, 31].

Some scholars have integrated DDSs into MNs, which improved the efficacy of melanoma treatment compared to the MNs and NTDDSs alone [32–34]. Wu et al. structured a redox-responsive nanomedicine based on disulfide-bridged degradable organosilica hybrid nanoparticles that were loaded with cisplatin and ethacrynic acid. The nanomedicine was further integrated into MNs for intralesional drug delivery against cisplatin-resistant melanoma [32]. Zhou et al. designed a polydopamine structured nano-shell to encapsule glucose oxidase for localized melanoma starvation therapy delivered by dissolving MNs [34]. These strategies achieved the effective inhibition of melanoma through enhanced topical delivery and combination therapy by integrating the DDSs into MNs. However, these nanomedicines, including triggerable nanomedicines, often suffer from drug leakage during topical administration or under non-triggering conditions [31]. In addition, the release of drugs from triggerable nanomedicines in the acidic and reducing tumor microenvironment is often insignificant, which significantly reduces the efficacy of the cancer therapy [32]. Therefore, achieving the controlled release/activation of drugs in tumors and guiding the treatment process through effective imaging techniques are necessary for safe and effective melanoma treatment [35, 36].

**Scheme 1:**
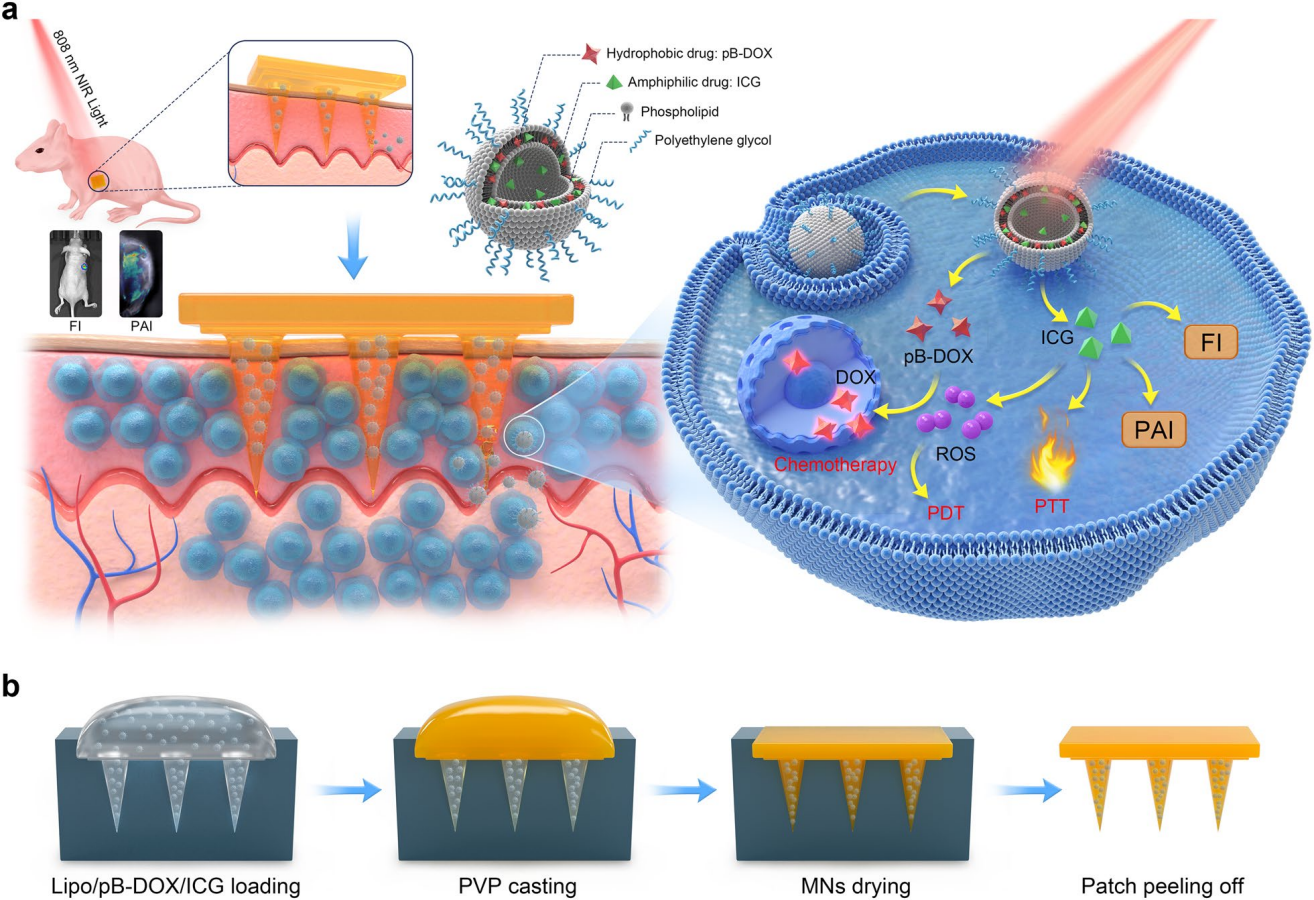
Design principle of the NIR light-activatable dissolving MN system (MN-pB/I) for multimodal theragnostic application in melanoma. **a** Schematic illustrations of the MN-pB/I. After directly injecting into the tumor sites, the matrix of the MN-pB/I quickly dissolved, releasing the Lipo/pB-DOX/ICG concentrated in the needle tips and distributing them evenly throughout the tumor tissue. The treatment regimens and efficacy could be guided and monitored by FI and PAI using the ICG photosensitizer. When exposed to NIR light, ROS produced by ICG performed PDT and activated pB-DOX for chemotherapy. The ICG can also exert PPT, as the high temperatures generated after irradiation promoted the dissolution of the MNs, as well as the permeation and cellular uptake of Lipo/pB-DOX/ICG, to effectively kill the melanoma cells. **b** Fabrication of the MN-pB/I patch: Lipo/pB-DOX/ICG loading, PVP casting, MNs drying, and patch peeling off

In this study, we combined the merits of MNs and DDSs to construct a near-infrared (NIR) light-activatable dissolving MN system for melanoma treatment (Scheme 1a). First, an ROS-responsive doxorubicin (DOX) prodrug (pB-DOX) was synthesized by incorporating a boronate moiety into the structure of DOX, which reduced the toxicity of the chemotherapeutic agent in normal cells and tissues until ROS activation. Then, liposomes were co-loaded with pB-DOX and the indocyanine green (ICG) photosensitizer [37]. The resulting Lipo/pB-DOX/ICG was dispersed into an aqueous solution of polyvinylpyrrolidone (PVP) in a MN mold, after which the solvent was evaporated under vacuum to prepare the dissolving MNs (MN-pB/I) containing Lipo/pB-DOX/ICG (Scheme 1b). After directly inserting the MNs locally into the tumor site on rats, the needle tips dissolved in the interstitial fluid, and the liposomes concentrated in the needle tips were released and distributed evenly throughout the tumor tissue. When the tumor sites were irradiated with NIR light, the photoexcited ICG generated ROS for photodynamic therapy (PDT) and activated pB-DOX for chemotherapy. Moreover, as a NIR dye, ICG was used to guide the treatment regimen and monitor the antitumor efficacy by fluorescence imaging (FI) and photoacoustic imaging (PAI).

## Experimental methods

### Materials

ICG was supplied by TCI (Tokyo, Japan). Doxorubicin hydrochloride (DOX·HCl) was supplied by Hisun Pharmaceutical (Zhejiang, China). Cholesterol was obtained from Hushi Chemical (Shanghai, China). Hydrogenated soybean phosphatidylcholine (HSPC) and 1,2-distearoyl-*sn*-glycero-3-phosphoethanolamine-*N*-[methoxy(polyethylene glycol)-2000] (DSPE-mPEG2000) were purchased from Avanti Polar Lipids (Alabaster, AL, USA). The Hoechst 33324 and MTT agent 3-(4,5-dimethylthiazol-2-yl)-2,5-diphenyltetrazolium bromide were purchased from Sigma-Aldrich (St Louis, MO, USA). PVP was purchased from Sinopharm Chemical Reagent (Shanghai, China). The Live/Dead viability/cytotoxicity kit was obtained from Invitrogen (Carlsbad, CA, USA). 2′,7′-Dichlorofluorescin diacetate (DCFH-DA) was obtained from Beyotime Institute of Biotechnology (Jiangsu, China). All solvents and chemicals were of analytical grade and used directly without further purification. Polydimethylsiloxane (PDMS) molds were purchased from Laike Mould firm (Guangzhou, China).

### Fabrication of Lipo/pB-DOX/ICG

The synthesis of pB-DOX and preparation of Lipo/pB-DOX/ICG were conducted using previously published methods [37].

### Fabrication and characterization of MN-pB/I

To prepare the blank MNs (B-MN), a 40% PVP K30 aqueous solution was poured into PDMS molds under vacuum, and the samples were dried overnight in a sealed desiccator, after which they were gently peeled off and stored in a desiccator. To prepare the Lipo/pB-DOX/ICG MNs patches (MN-pB/I), the Lipo/pB-DOX/ICG nanoliposomes were first dispersed into the PDMS MNs molds under vacuum. After deposition under vacuum, the solvent of the nanoliposomes was allowed to evaporate over a period of 30 min to concentrate the Lipo/pB-DOX/ICG at the MNs tips. The redundant solution was removed to ensure that each cavity was filled with the same volume of liposomes before filling the PVP K30 (40%) matrix to form the base. Finally, the MN-pB/I patches were gently peeled from the PDMS mold and placed in a dryer at 4 °C in the dark until use. The MN patches containing Lipo/pB-DOX (MN-pB) or Lipo/ICG (MN-I) only were prepared in a similar manner. The morphologies of MN-pB/I were observed with a stereo microscope (VS-1602T, Shanghai, China), an inverted fluorescence microscope (Model IX71 Olympus, Tokyo, Japan), and SEM (S-3400N, Hitachi, Japan).

To quantify the Lipo/pB-DOX/ICG loading, the needles of the prepared MN-pB/I patches were scraped off and dissolved in 0.5 mL chloroform, and the solution was ultrasonicated and centrifuged (12,000 rpm, 3 min) to collect the supernatant. The loading contents (LC) of pB-DOX in MN-pB/I was determined using a previously published high-performance liquid chromatography (HPLC, Agilent, Santa Clara, USA) assay [37].

A tensile load frame was used to measure the mechanical strength of the MN-pB/I patch. The tensile force was consecutively monitored by a stress–strain gauge as a stainless-steel plate compressing arrays of MNs along the *y*-direction. The initial gauge between the stainless-steel plate and the MN tips was set at 2.00 mm, and the stainless-steel plate top was moved toward the MN-array patches at a speed of 0.1 mm/s. When the needles began to buckle, the failure force of MNs was recorded.

To study the in vitro dissolution and drug release behavior of the MNs, the MN-pB/I patches were inserted into the hairless skin of Sprague–Dawley (SD) rats and removed, and the height of the needle tips was determined using an inverted fluorescence microscope (Model IX71 Olympus, Tokyo, Japan) 1, 2, 3, and 5 min after removal of the patch. The residual drug on the skin was wiped off with cotton swabs and dissolved in chloroform. Then, the remaining MNs needles were scraped off and dissolved in the same chloroform solution. The recovered drug content in the chloroform was determined by HPLC analysis. The drug content entering the skin was calculated indirectly by subtracting the recovered drug content from the original LC.

To assess the in vitro skin penetrability of the MN-pB/I, the MNs patches were inserted into the dorsal skin of SD rats for 5 min until the needle array was completely dissolved in the skin. Then, the skin was observed under a confocal laser scanning microscope (CLSM, Leica, Germany), and images were taken with a camera. The insertion ratio of MN-pB/I was equal to the number of points on the skin surface divided by 100 (the number of tips per MNs patch). The depth at which MNs penetrated the skin was measured on the frozen tissue sections. The recovery of the skin after MN-pB/I treatment was investigated on the back skin of BALB/c nude mice. The MNs patches were inserted into the skin by thumb and left for 5 min. After removing the patches, the changes of skin morphology were observed by camera at different timepoints.

### Cell lines

A375 cells were obtained from Xiangya Central Experiment Laboratory (Hunan, China) and cultured in Dulbecco’s Modified Eagle’s Medium supplemented with 10% (v/v) fetal bovine serum and 1% (v/v) penicillin–streptomycin in a humidity-controlled atmosphere containing 5% CO_2_ at 37 °C.

### Cellular uptake of Lipo/pB-DOX/ICG

A375 cells were incubated overnight in CLSM dishes at a concentration of 2 × 10^5^ cells per well. The cells were treated with PBS, DOX·HCl, Lipo/ICG, Lipo/pB-DOX, and Lipo/pB-DOX/ICG at an identical ICG (3.0 μg/mL) and/or pB-DOX (6 μg/mL) concentration for different periods of time. Subsequently, the cells were stained with Hoechst 33334 for 20 min, rinsed twice with PBS and observed under a CLSM. After incubating in a humidified atmosphere containing 5% CO_2_ at 37 °C for different time periods (0, 2, 12, and 24 h), the cells were collected and analyzed by flow cytometry (FACSVerse, BD, USA) to quantitatively measure the fluorescence intensity emitted from the liposomes in the cells.

### Effect of Lipo/pB-DOX/ICG on intracellular ROS levels

Intracellular ROS levels after incubating cells with Lipo/pB-DOX/ICG were measured using the DCFH-DA fluorescent probe, which can be rapidly oxidized into DCF with green fluorescent in the presence of ROS. Briefly, A375 cells were seeded in 12-well plates (2 × 10^5^ per well) and incubated overnight. The ROS inhibitor NaN_3_ (100 mM) [38] was added to the cell suspensions, and the plates were incubated for another 12 h, depleting the original ^1^O_2_ produced by A375 cells themselves. Then, the medium in each well was replaced with 1 mL of fresh culture medium containing either PBS, Lipo/ICG, Lipo/pB-DOX, or Lipo/pB-DOX/ICG (the concentration of ICG was 5.0 μg/mL). After incubating for another 2 h, the cells were washed thrice with PBS and cultured in 1 mL DCFH-DA (25 μM) for 20 min. Finally, each well was washed twice with PBS and irradiated (808 nm, 1.0 W/cm^2^) for different periods of time, and the fluorescence intensity of DCF (λ_ex_ = 495 nm, λ_em_ = 529 nm) was immediately measured by an inverted fluorescence microscope. To analyze the fluorescence intensity quantitatively, flow cytometry was performed using the method described above.

To study the effect of ROS on the activity recovery of pB-DOX, the A375 cells were subjected to 808 nm laser irradiation at 1 W/cm^2^ for different periods of time after incubating the cells with Lipo/pB-DOX/ICG for 2 h, and the fluorescence intensity of pB-DOX/DOX was determined by CLSM and flow cytometry.

### In vitro cytotoxicity of Lipo/pB-DOX/ICG

The in vitro cytotoxicity of Lipo/pB-DOX/ICG were assessed in A375 cells by MTT assays. First, A375 cells (1 × 10^4^ cells per well) were seeded into 96-well plates and incubated overnight in a humidified atmosphere containing 5% CO_2_ at 37 °C. Then, the cells were treated with different liposomal formulations (Lipo/pB-DOX, Lipo/ICG + laser, Lipo/pB-DOX/ICG, and Lipo/pB-DOX/ICG + laser) at different concentrations in fresh culture medium. After incubating for 6 h, the cells were irradiated with 808 nm laser at 1.0 W/cm^2^ for 2 min. 24 h later, the MTT solution (5 mg/mL, 20 μL) was added to the cell solutions, which were incubated for another 4 h. Finally, cell viability was evaluated using a microplate reader (Infinite M200 PRO, TECAN, Austria) after reacting with 100 μL DMSO under mild shaking at 37 °C.

To visualize the phototherapeutic efficacy of the Lipo/pB-DOX/ICG, including photothermal therapy (PTT) and PDT, A375 cells irradiated with the 808 nm laser were immediately washed thrice with PBS, stained with calcein-acetoxymethyl (AM, used to stain live cells) and propidium iodide (PI, used to stain dead cells) according to the instructions of the kit. Then, the cells were trypsinized, resuspended in 1.0 mL PBS, and observed under an inverted fluorescence microscope. To further evaluate the cytotoxicity of the drug-loaded liposomes, the A375 cells that were irradiated with an 808 nm laser (1.0 W/cm^2^) were incubated for another 12 h. Then, A375 cells were immediately washed thrice with PBS and stained with calcein-AM/PI. Then, the living/dead cells were trypsinized, resuspended in 1.0 mL PBS, and observed under an inverted fluorescence microscope, and the fluorescence intensity in the cells was quantitatively analyzed by flow cytometry.

### Tumor models

BALB/c nude mice (female, 4–5 weeks) were utilized for developing a melanoma tumor model. The mice were acclimated to standard diet at 25 °C for 1 week prior to the study. A375 cells in 100 μL of serum-free medium (2 × 10^6^) were injected subcutaneously into the right forelimb of the mice to establish the melanoma tumor-bearing model. The tumor volume was calculated as follows: tumor volume = length × (width)^2^/2.

### In vivo FI and PAI of MN-pB/I

When the tumor size was greater than 200 mm^3^, the B-MN, MN-I, and MN-pB/I patches were inserted into tumor sites of the mice bearing A375 tumors. A Xenogen IVIS Spectrum imaging system (Perkin Elmer, USA) was used to measure the in vivo fluorescence intensity of ICG (Ex: 735 nm/Em: 805 nm) 0.5 h following insertion of the patches. Several mice were sacrificed for ex vivo imaging of the major organs (hearts, livers, spleens, lungs, and kidneys) and tumor tissues. For the PAI, photoacoustic (PA) signals of tumor-bearing mice were monitored by a VEVO LASER PAI system (VEVO 2100, FUJIFILM Visual Sonics, INC, USA).

### In vivo antitumor efficacy of MN-pB/I

When the tumor volumes reached about 150–200 mm^3^, the tumor-bearing mice were randomly divided into six groups (six mice per group): B-MN, B-MN + laser, MN-pB, MN-I + laser, MN-pB/I, and MN-pB/I + laser. The patches were inserted into the tumor sites, and the mice in the laser groups were irradiated after 0.5 h post-insertion with an NIR laser for 2 min (1 W/cm^2^). During the laser irradiation periods, the temperature changes within the tumors were monitored using an infrared thermal imaging camera (FLIR E50; Estonia). The tumor volumes and body weights were recorded every 2 days for 15 days. The tumor growth curves were generated by plotting the average tumor volume vs. the days after the first treatment. Fifteen days later, all mice were sacrificed, and their tumors were resected and weighed. Then, the inhibition rate of tumor growth (IRT) was calculated using the following equation: IRT = 100% × (mean tumor weight of the PBS group − mean tumor weight of the experimental group)/mean tumor weight of the PBS group × 100%.

### Statistical analysis

The statistical analysis (*t*-test) was carried out in OriginPro 9.0 (OriginLab, MA, USA). All data reported herein represent the mean ± standard deviation (SD). *p < 0.05 was considered statistically significant. (NS, no significance, *p < 0.05, **p < 0.01, ***p < 0.001, ****p < 0.0001).

## Results and discussion

### Fabrication and characterization of MN-pB/I

**Fig. 1:**
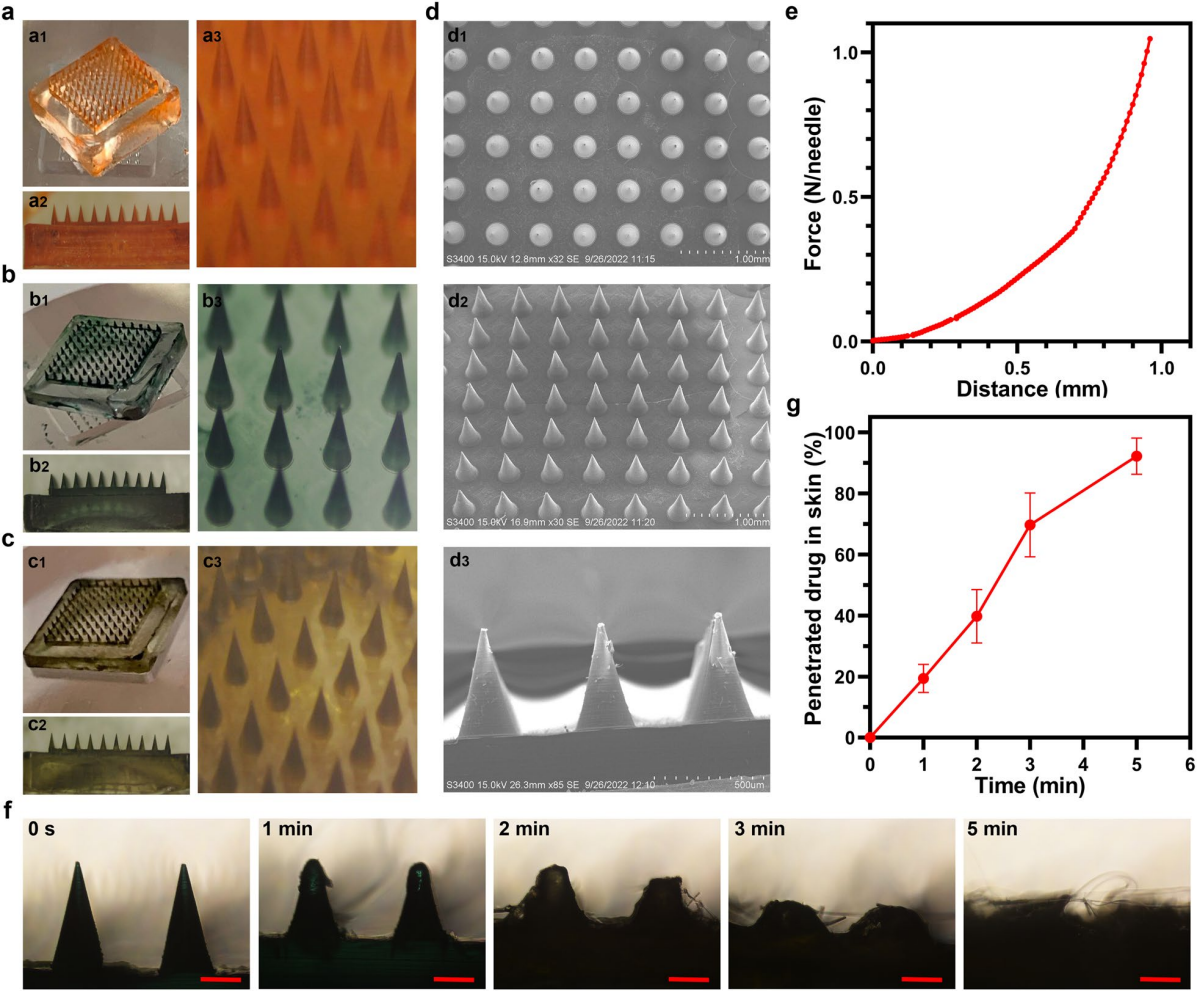
Characterization of the MNs. Bright-field micrographs of MN-pB (**a**_**1**_–**a**_**3**_), MN-I (**b**_**1**_–**b**_**3**_), and MN-pB/I (**c**_**1**_–**c**_**3**_): **a**_**1**_, **b**_**1**_, and **c**_**1**_, general views of MNs patches; **a**_**2**_, **b**_**2**_, and **c**_**2**_, front views of MNs patches; **a**_**3**_, **b**_**3**_, and **c**_**3**_, high magnification views of MNs patches. **d** SEM images of MN-pB patch in drying state: **d**_**1**_–**d**_**3**_ general view, front view, and high magnification view of MN-pB patch. **e** Mechanical properties of MN-pB/I. **f** Morphology of MN-pB/I after inserting into the skin for different time points. **g** The penetrated drug content in skin after the insertion of MN-pB/I at different time points. The scale bar is 200 μm

In this study, we incorporated the Lipo/pB-DOX/ICG into dissolving MNs for in vivo administration. MN-pB/I was prepared using PVP as the matrix because of its excellent biocompatibility, tailored cross-linking density, and strong mechanical properties [39]. Using the commercial MNs molds, MN-pB, MN-I, and MN-pB/I were fabricated with uniform size and morphology (Figs. 1a–d). A 10 × 10-needle array was assembled on a 0.81 × 0.81 cm^2^ patch with a center-to-center interval of 600 μm, and liposomes were concentrated at the tips of the needles through multiple depositions. Each needle featured a conical shape that was 600 μm in height and 300 μm in diameter at the base and had a sharp tip tapering to a 5 μm radius of curvature, which was accurately formed using the master mold. Compared to the blank MNs (B-MN), MN-pB, MN-I, and MN-pB/I were red-, green-, and brown-colored, respectively, in the needle tips, indicating that that liposomes were concentrated at MNs tips. To quantify the Lipo/pB-DOX/ICG loading within the MNs, the needles of the MN-pB/I patches were scraped off, and the LC of pB-DOX in the needles was determined by HPLC using a previously published assay [37]. Based on HPLC analysis, each MN patch was calculated to contain 5.03 μg of pB-DOX.

Compared to systemic administration approaches, transdermal drug delivery methods have attracted extensive attention in melanoma treatment because the drugs can avoid first-pass metabolism and exert fewer off-target effects [29, 30]. However, the SC regulates transdermal drug delivery by acting as a barrier to prevent the penetration of most drugs across the skin, thereby leading to ineffective absorption [32, 33]. MNs can overcome this issue by non-invasively piercing the SC by mechanical force [34]. Therefore, we conducted mechanical compression tests on the MN patches (Fig. 1e). The force acting on the MNs continuously increased with increasing displacement of MNs, reaching 1.05 N/needle with a displacement value of 0.96 mm, which was sufficient enough for the needle to pierce the skin without breaking.

Since the dissolution of the matrix within the MNs directly affects the release of the Lipo/pB-DOX/ICG, we studied the dissolution behavior of MN-pB/I in vitro. As shown in Fig. 1f, the height of the needle tips decreased by approximately 30% 1 min after insertion of the MN-pB/I patch into the tissue, followed by approximately half within 2 min. After 5 min, the MNs were almost completely dissolved, and only part of villi were attached to the PVP base. Because of the low drug content in the single MN-pB/I patch, the indirect method was used in this study instead of the traditional Franz diffusion cell to evaluate the in vitro transdermal drug release. As shown in Fig. 1g, the drug content in the skin reached 39.74% after 2 min. After 5 min, the needles were completely dissolved, and the drug content in skin reached 92.20%. These results indicated that not only did the MN-pB/I have excellent solubility, but also the Lipo/pB-DOX/ICG could be easily and efficiently released from the PVP matrix to transdermally deliver drugs.

**Fig. 2:**
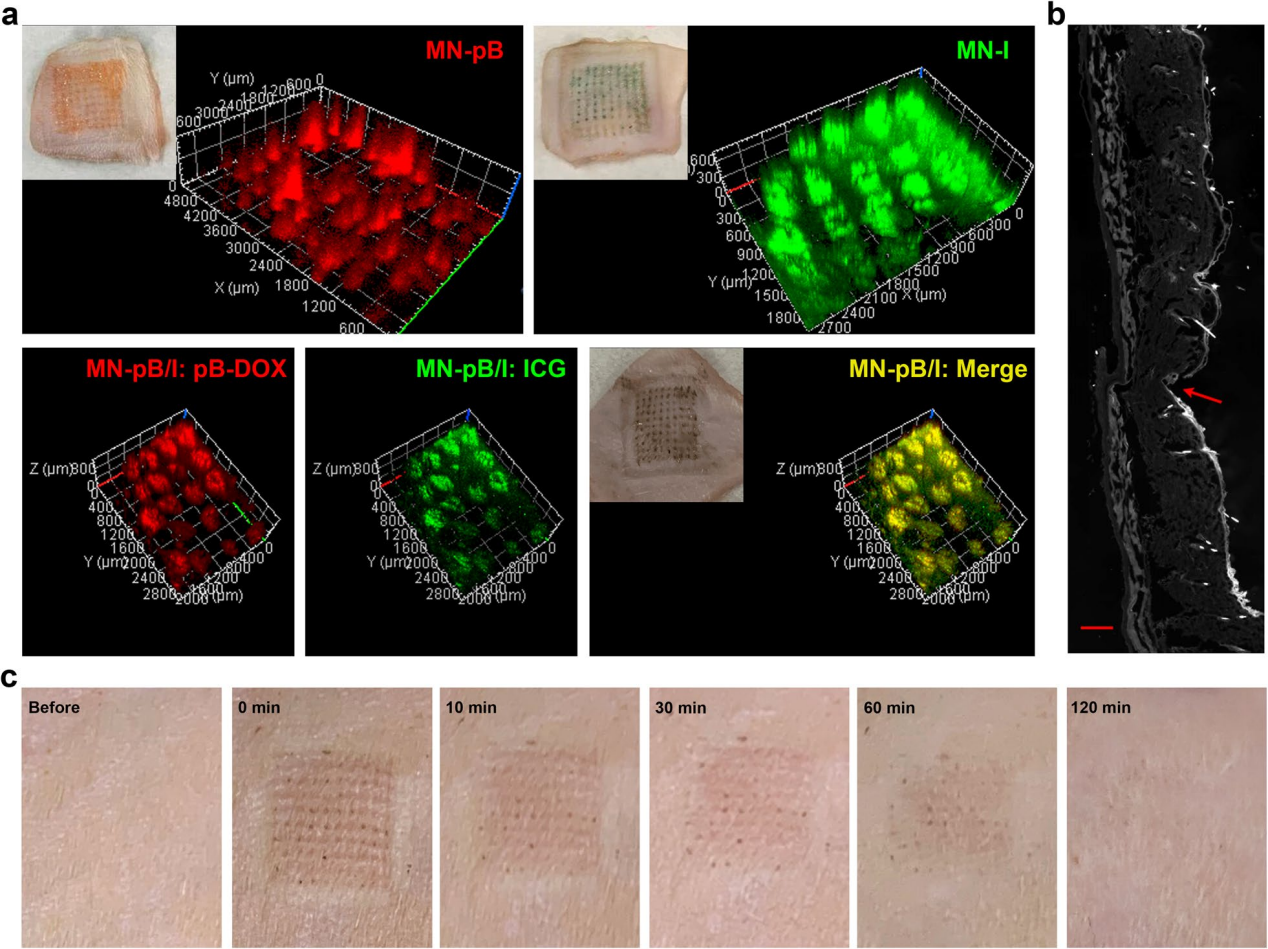
**a** Confocal 3D reconstruction images of the skin of SD rats punctured with MN-pB, MN-I, and MN-pB/I patches (insets: pictures of the skin of SD rats punctured with MNs). **b** Image of frozen tissue sections to observe the cross-sectional area of the mouse skin (scale bar: 200 μm). **c** The appearance of the skin before and after treatment with the MNs at different time points

To directly assess the strength of the MNs, the MNs were inserted into the dorsal skin of SD rats, and the skin insertion ratios were evaluated (Fig. 2a). The 100-needle arrays were easily inserted into the SD rat skin using the gentle force of a thumb, and the insertion ratio of MN-pB/I was calculated to be 97% based on the color of the matrix on the skin. After histological examination, the insertion depth of the MN-pB/I was approximately 300 μm (Fig. 2b), indicating that the MNs successfully crossed the SC and penetrated into the epidermis and dermis. Even though the MNs were approximately 600 μm in length, they only reached the dermis and did not pierce the skin, possibly due to skin elasticity as well as the rapid dissolution of the MNs matrix during the insertion process [8, 31]. To directly assess the permeability of the loaded drugs, the skin of the SD rats punctured with the MNs was observed by CLSM. The fluorescence depth extended below 300 μm in the skin tissue samples punctured by MN-pB, MN-/I, and MN-pB/I, and the overlap of the green and red fluorescence indicated that both pB-DOX and ICG in MN-pB/I crossed the SC and penetrated the dermis (Fig. 2a). When the tips of the MNs were inserted into the skin of the SD rats, PVP could absorb fluid from the surrounding tissue due to its excellent hygroscopicity [39]. Upon insertion, dissolution occurred, allowing the drugs to penetrate deeper than the insertion depth. The CLSM results showed that the MN-pB/I could achieve the rapid, transdermal delivery of drugs (Fig. 2a).

Next, we investigated the biocompatibility of MN-pB/I by evaluating how the skin recovered after treatment. The patch was inserted into the back skin of the BALB/c nude mice and peeled off 5 min later. At 60 min post-insertion, the majority of the erythema had faded, and the micro-pathways created by the needles had been resealed (Fig. 2c), avoiding the risk of pathogen infection. After 120 min, the skin essentially recovered to the original condition, and no abnormalities or other adverse reactions of the experimental mice were observed in the following days. Due to the small size of the needle tips as well as the fast dissolution of the matrix, MN-pB/I was minimally invasive to the skin, making the MNs highly compliant for patient administration.

### Cellular uptake of Lipo/pB-DOX/ICG

**Fig. 3:**
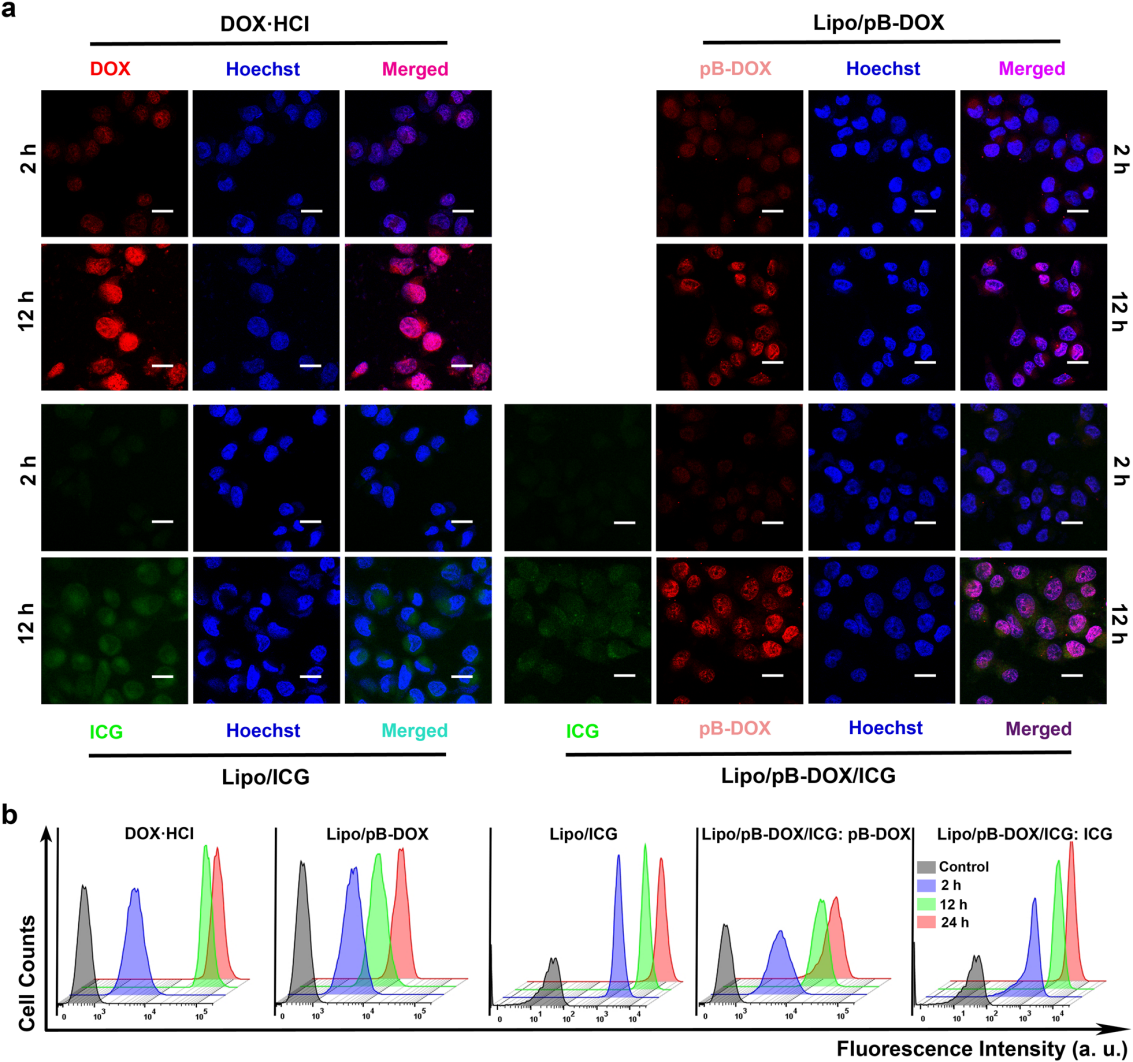
**a** CLSM images of A375 cells incubated with DOX·HCl, Lipo/pB-DOX, Lipo/ICG, and Lipo/pB-DOX/ICG for 2 and 12 h (scale bar: 50 μm). **b** Flow cytometry analysis of A375 cells incubated with different treatment groups for different incubation periods. (Ex: 735 nm/Em: 805 nm for ICG; Ex: 505 nm/Em: 550 nm for pB-DOX)

After confirming that MN-pB/I could penetrate the SC and effectively release Lipo/pB-DOX/ICG in the underlying tissue, we investigated the in vitro efficacy of Lipo/pB-DOX/ICG. The subcellular drug distribution and release in A375 cells treated with DOX·HCl, Lipo/ICG, Lipo/pB-DOX, and Lipo/pB-DOX/ICG were observed by CLSM. Figure 3a shows that the red fluorescence (from DOX or pB-DOX) of DOX·HCl, Lipo/pB-DOX, and Lipo/pB-DOX/ICG was mainly distributed in the nucleus, and the fluorescence intensity increased over time until 12 h, after which no fluorescence increase was observed. Since DOX acts on DNA and topoisomerase II, both of which are located in the nucleus, the accumulation of DOX in the nucleus is crucial for inducing apoptosis [40]. In addition, Lipo/pB-DOX and Lipo/pB-DOX/ICG exhibited lower red fluorescence intensities than DOX·HCl both after 2 h and 12 h, which was attributed to the inadequate intracellular activation of pB-DOX that resulted in a weaker fluorescence intensity. The green fluorescence (from ICG) of Lipo/pB-DOX/ICG and Lipo/ICG also increased over the incubation period. Unlike pB-DOX (or DOX), ICG was mainly distributed in the cytoplasm after incubation for 2 h and 12 h, and this subcellular distribution did not affect its therapeutic efficacy. Flow cytometry was employed to quantitate the cellular uptake of DOX·HCl and the liposomal NPs (Fig. 3b). The fluorescence intensities of both pB-DOX (or DOX) and ICG were directly proportional to the incubation time over the 12 h incubation period, indicating that the liposomal NPs were gradually being taken up by the A375 cells. However, after 12 h, the fluorescence intensities did not increase significantly, indicating that the cellular uptake of the liposomal NPs reached saturation.

### Intracellular ROS detection of Lipo/pB-DOX/ICG

**Fig. 4:**
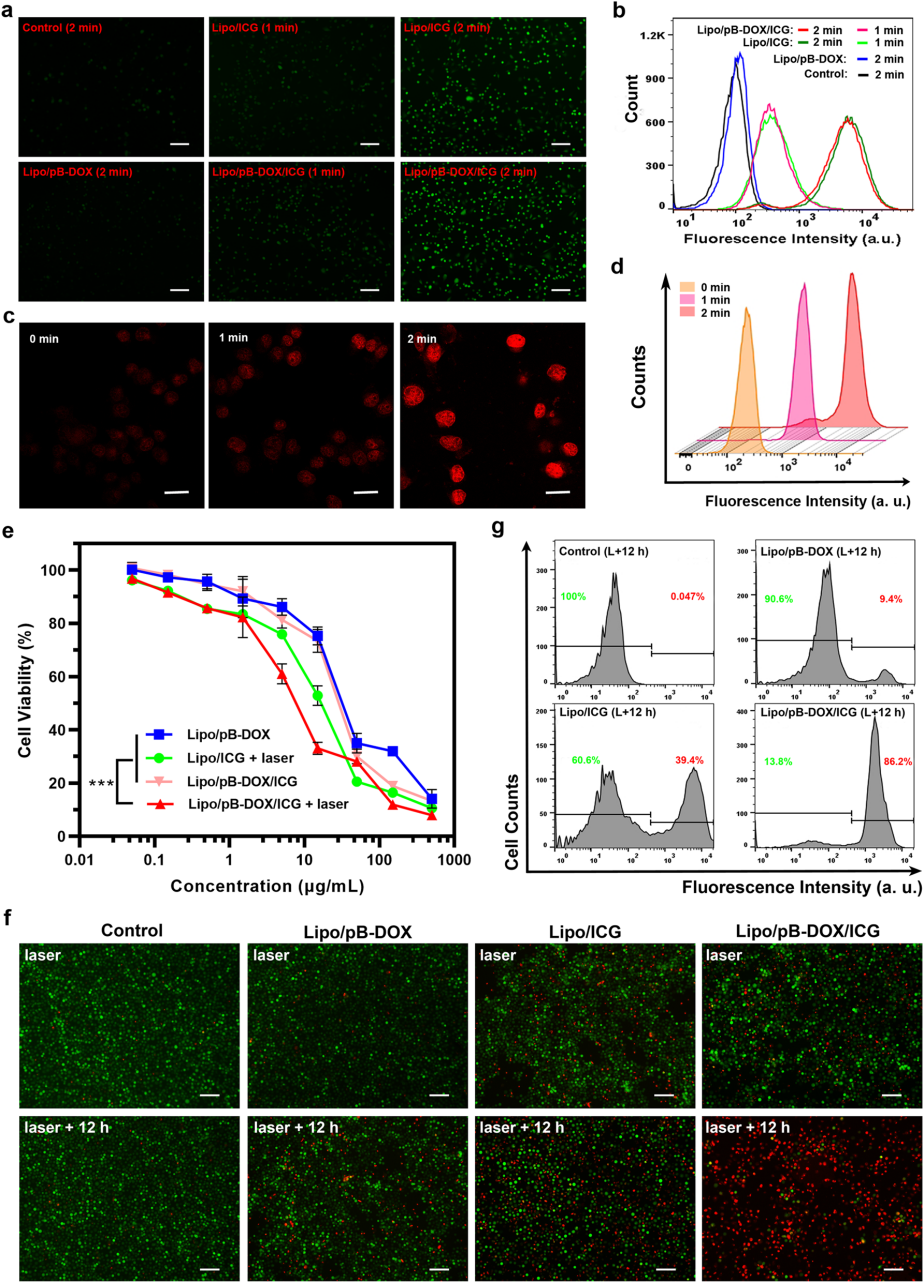
**a** Fluorescence images of A375 cells containing Lipo/pB-DOX, Lipo/ICG, and Lipo/pB-DOX/ICG after intracellular ROS generation by laser irradiation (1.0 W/cm^2^) for 1 or 2 min (scale bar: 200 μm). **b** Flow cytometry analysis of A375 cells after different treatments. **c** CLSM images of A375 cells incubated with Lipo/pB-DOX/ICG and irradiated for different periods of time (scale bar: 50 μm). **d** Flow cytometry analysis of A375 cells incubated with Lipo/pB-DOX/ICG and irradiated for different periods of time. Ex: 488 nm/Em: 525 nm for DCF; Ex: 505 nm/Em: 550 nm for pB-DOX. **e** MTT assays of Lipo/pB-DOX, Lipo/ICG + laser, Lipo/pB-DOX/ICG, and Lipo/pB-DOX/ICG + laser groups over 24 h in A375 cells. **f** Fluorescence images of A375 cells co-stained with PI and AM and treated under the different conditions listed in **e** after incubation for 0 or 12 h (overnight) and NIR irradiation (scale bar: 100 μm). The live cells are stained green, and the dead cells are stained red. **g** Quantitative flow cytometry analysis of the A375 cells stained with PI. The data shown represent the mean ± SD, n = 3 per group. *NS* no significance. *p < 0.05, **p < 0.01, ***p < 0.001, ****p < 0.0001)

Pretreated with NaN_3_ to scavenge endogenous ^1^O_2_ in the A375 cells, the intracellular ROS produced by Lipo/pB-DOX/ICG under sustained laser irradiation was detected using an ROS-sensitive probe DCFH-DA. As shown in Fig. 4a, the green fluorescence (from DCF) of PBS (control) and Lipo/pB-DOX groups were negligible after 2 min of laser irradiation. In contrast, the cells incubated with Lipo/ICG and Lipo/pB-DOX/ICG both produced apparent green fluorescence within 1 min of laser irradiation. After 2 min, both the number of the fluorescent cells and the intensity of the fluorescence increased significantly. The flow cytometry results (Fig. 4b) showed that the intensity increased during the irradiation period, which was consistent with Fig. 4a. This indicated that ROS were significant produced in the presence of Lipo/pB-DOX/ICG inside the tumor cells under irradiation, which will play an indispensable role in PDT and subsequent initiation of chemotherapy.

The fluorescence emission from pB-DOX is inherently lower than its parent compound DOX. However, the reduced fluorescence emission can be recovered by converting the prodrug to DOX by ROS. Therefore, to verify that the ROS produced by ICG is able to induce intracellular DOX activation, the cells incubated with Lipo/pB-DOX/ICG for 2 h were irradiated with an 808 nm laser at 1 W/cm^2^ for different periods of time, and the change in the intensity of the red fluorescence (from DOX or pB-DOX) in the cells was observed by CLSM and qualitatively analyzed by flow cytometry. As shown in Fig. 4c, d, the fluorescence intensity of the Lipo/pB-DOX/ICG inside the cells increased significantly after 1 min of irradiation, and the increase was directly proportional to the irradiation time, indicating that more and more pB-DOX transformed into DOX. Overall, these results indirectly indicated that the ROS produced by ICG after NIR irradiation could successfully convert pB-DOX into DOX.

### In vitro cytotoxicity of Lipo/pB-DOX/ICG

The cytotoxicity of Lipo/pB-DOX/ICG at different pB-DOX and ICG concentrations was quantitatively assessed using MTT assays. A375 cells were treated with Lipo/ICG + laser, Lipo/pB-DOX, Lipo/pB-DOX/ICG, and Lipo/pB-DOX/ICG + laser, and the cell viabilities of each group decreased with increasing drugs concentrations (Fig. 4e). The half maximal inhibitory concentration (IC_50_) of Lipo/ICG + laser, Lipo/pB-DOX, Lipo/pB-DOX/ICG, and Lipo/pB-DOX/ICG + laser were 15.81, 30.40, 25.98, and 7.52 μg/mL (calculated by pB-DOX concentration), respectively. Based on the IC_50_ values, Lipo/pB-DOX was the least toxic liposomal formulation, while Lipo/pB-DOX/ICG + laser was the most efficacious toward killing the cancer cells compared to other groups. Because the ROS concentration of the tumor microenvironment is low, the activation of pB-DOX on A375 cells was insufficient. Therefore, MN patches containing Lipo/pB-DOX and Lipo/pB-DOX/ICG (without laser) were far less toxic than those containing Lipo/pB-DOX/ICG (with laser). Under laser irradiation, pB-DOX was activated, enabling the Lipo/pB-DOX/ICG to exert a synergistic phototherapeutic and chemotherapeutic effect.

To visualize the phototherapeutic efficacy of Lipo/pB-DOX/ICG, a live/dead cell co-staining assay was performed. As shown in Fig. 4f, there were almost no red fluorescence-stained cells in the PBS and Lipo/pB-DOX groups, while the images of the Lipo/ICG and Lipo/pB-DOX/ICG groups featured some red fluorescent cells. These results indicated that Lipo/pB-DOX/ICG not only converted the NIR light energy into local hyperthermia for PTT but also produced additional ROS for PDT, demonstrating that the liposomal particles exhibited a phototherapeutic effect after irradiation with light.

To further evaluate the synergistic phototherapeutic and chemotherapeutic effect of the loaded liposomes, the A375 cells were incubated for 12 h after irradiation at 808 nm for 2 min. The Lipo/ICG and Lipo/pB-DOX/ICG groups featured more red fluorescent cells than the groups that did not undergo the 12 h incubation period, and the cell death rates of the two groups reached 39.4% and 86.2% (Fig. 4g), respectively. However, the cell death rates of the PBS and Lipo/pB-DOX groups that underwent the 12 h incubation were only 0.05% and 9.4%, respectively. After incubation overnight, pB-DOX was gradually converted into DOX by the in situ-generated ROS, which subsequently entered the nucleus for chemotherapy. Based on the data in Fig. 4f, g, we concluded that the synergistic phototherapeutic and chemotherapeutic effect was more efficacious than the phototherapeutic effect alone.

### In vivo FI and PAI of MN-pB/I

**Fig. 5:**
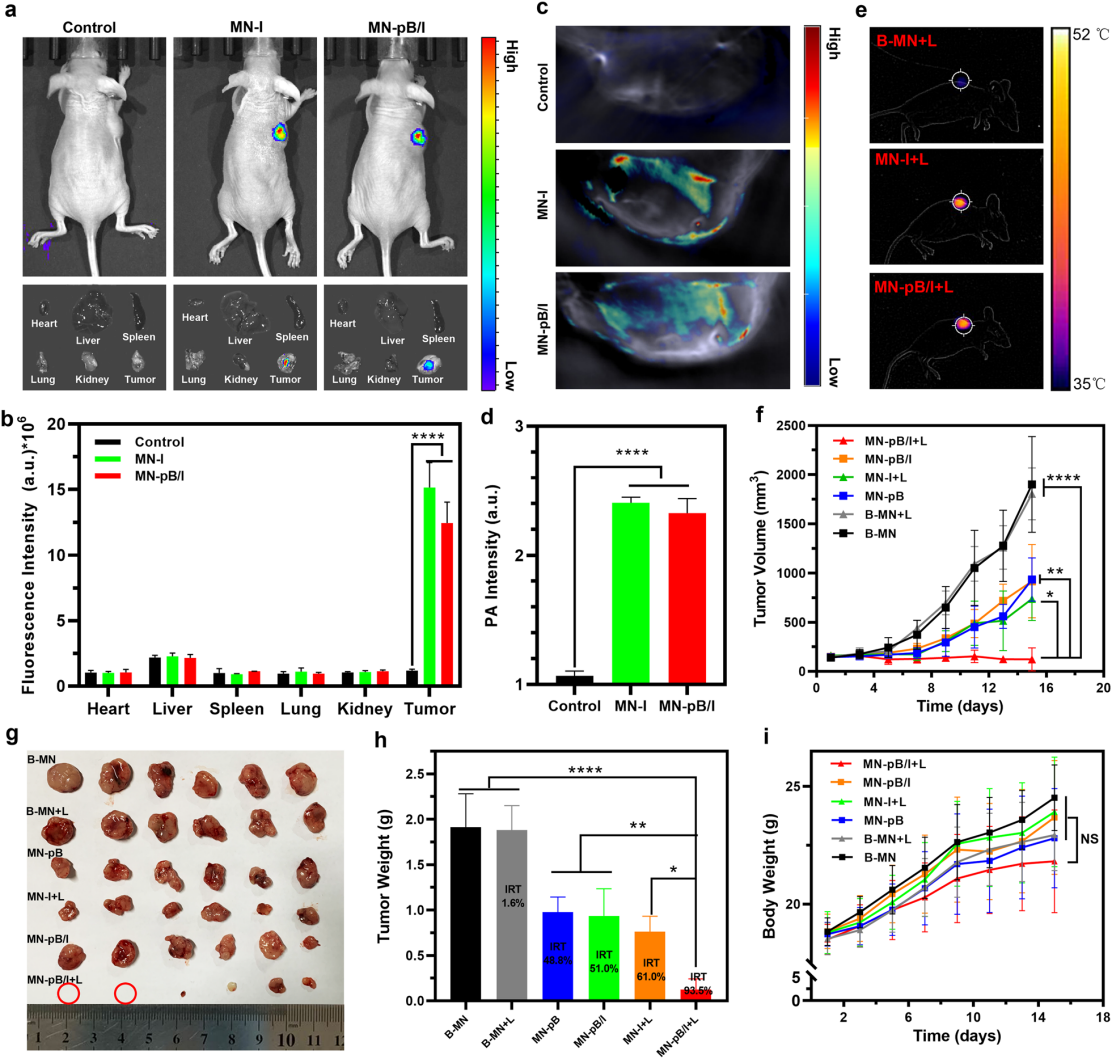
**a** Fluorescence images of mice bearing A375 tumors and ex vivo fluorescence images of the major organs and tumors dissected from the mice after being treated with B-MN (control), MN-I, and MN-pB/I patches. **b** Semiquantitative biodistribution of nanoliposomes in the mice determined by averaging the fluorescence intensities from the images of the organs and tumors. **c** PA images of the tumor sites on the A375 tumor-bearing mice treated with the B-MN, MN-I, and MN-pB/I patches. **d** The corresponding PA signals at the tumor sites of the A375 tumor-bearing mice. **e** Thermal images of the A375 tumor-bearing mice from different treating groups (B-MN + laser, MN-I + laser, and MN-pB/I + laser) under 808 nm laser irradiation for 2 min. **f** Tumor volume curves of the different mice groups following treatment with B-MN, B-MN + laser, MN-pB, MN-I + laser, MN-pB/I, and MN-pB/I + laser. **g** Photographs of the tumor tissues removed from the groups treated with different formulations after 15 d. The red circles indicated that the tumors had disappeared. **h** Tumor weights of each group at the end of the experiment and IRT. **i** Body weight curves of the A375 tumor-bearing mice in each group. The data shown represent the mean ± SD, n = 3 per group. *NS* no significance. *p < 0.05, **p < 0.01, ***p < 0.001, ****p < 0.0001

ICG has been widely used as a fluorescence and PA contrast agent due to its strong optical absorbance in the NIR region [41, 42]. FI and PAI are both nonradioactive imaging techniques. FI provides a unique approach to monitor the dynamic distribution of contrast-containing NPs throughout the whole body [41], while PAI can visualize the accumulation of NPs within the deep tumor region with improved imaging resolution [42]. When the tumor size of the tumor-bearing mice reached about 200 mm^3^, the B-MN, MN-I, and MN-pB/I patches were inserted into the skin at the tumor site and monitored by in vivo NIR imaging. Unlike the drug-free B-MN, considerable fluorescence signals from the MN-I and MN-pB/I patches were observed in the tumors on the mice (Fig. 5a). To confirm that the nanoliposomes penetrated the tumor, the tumor-bearing mice were sacrificed, and the tumors and major organs were excised for FI. Ex vivo images (Fig. 5a) showed bright fluorescence signals in the tumor tissue of the mice treated with MN-I and MN-pB/I, and there was no observable fluorescence signal in the major organs, indicating that the NIR light-activatable system rapidly dissolved in the skin without drug leakage. Figure 5b displays the fluorescence intensities of the ex vivo fluorescence images of the major organs and tumor tissues after quantitative analysis. The fluorescence intensities of the MN-pB/I in the major organs of all treatment groups were very low, while the corresponding fluorescence intensities in the tumors dissected from the mice in the MN-pB/I group were an average of 9.49 times higher than that of the control group. Subsequently, the in vivo PAI of MN-pB/I was conducted using a Vevo LAZR PAI system [40]. Unlike B-MN, considerable PA signals were observed in the tumors of the mice bearing the MN-I and MN-pB/I patches (Fig. 5c, d), which was consistent with the FI results in Fig. 5a, b. The PA signals in the tumors derived from the mice in the MN-pB/I group was 9.96 times higher than that of the control group. These results suggested that Lipo/pB-DOX/ICG could effectively accumulate at the tumor sites and achieve targeted therapy after administration via the MNs.

### In vivo antitumor efficiency of MN-pB/I

The photothermal performance of MN-pB/I and the dual-modal imaging-guided tumor therapy were subsequently investigated in vivo. A375 tumor-bearing mice were treated with MN-pB/I or other control MNs patches, and the tumor sites were irradiated with an 808 nm laser (1.0 W/cm^2^) at 0.5 h post-injection. In vivo thermal images were taken using an infrared thermal imaging camera. The thermal images show that the temperature of tumor regions in MN-pB/I + laser group rapidly reached about 46.2 °C (Fig. 5e). Compared to the B-MN + laser group, the temperature within the tumor in the MN-pB/I + laser group increased more rapidly, demonstrating that the liposomal particles in the MN-pB/I patch were capable of undergoing PTT upon laser irradiation to kill the tumor cells after permeation of the tumor by the Lipo/pB-DOX/ICG. The elevated temperatures also increased the solubility of the MNs in the tumor tissue matrix and the permeation and cellular uptake of the liposomes.

To evaluate the synergistic phototherapeutic and chemotherapeutic effect of the DSS-loaded MNs, the changes in tumor volumes and body weights of the tumor-bearing mice were recorded every 2 days after each treatment. During the treatment, compared to the B-MN and B-MN + laser groups, tumor growth inhibition was displayed in all drug-treated groups. However, the tumors could only be partly inhibited by MN-pB, MN-I + laser, and MN-pB/I groups because of their different deficiencies. The tumor volumes in the MN-pB/I + laser group were significantly reduced and were even completely ablated after a single dose, indicating that this MN patch exhibited the best tumor suppression efficacy (Fig. 5f). The visualized photographs of the tumor tissues treated with different formulations and conditions are shown in Fig. 5g, and the results were consistent with the relative tumor volume change curves shown in Fig. 5f. Based on these data, the calculated IRT for the MN-pB/I + laser group was 93.5%, which was significantly higher than the MN-pB group (48.8%), MN-pB/I group (51.0%), and MN-I + laser group (61.0%) (Fig. 5h). The body weight change curves (Fig. 5i) of the tumor-bearing mice were also used to assess the therapeutic efficacy and toxicity of the different treatments. Due to the excellent biocompatibility of MN-pB/I, the body weight of the tumor-bearing mice steadily increased over the 15-day period, while the tumor volume decreased, and no treatment-induced adverse reactions were observed during the treatment.

## Conclusion

In this study, a photoactivatable, dissolving MN system (MN-pB/I) was prepared by assembling a PVP MN array with liposomes co-loaded with ROS-responsive pB-DOX and ICG. The combination of DDSs and MNs not only enables the delivery of poorly soluble or insoluble drugs by MNs but also overcomes the difficulty of NTDDSs to penetrate the skin and to distribute the drugs intratumorally during the melanoma therapy. Moreover, the externally controlled NIR irradiation and the activation of the prodrug pB-DOX by ROS generated by irradiation jointly realize the intratumoral controllable drug release/activation. ICG can be used as a fluorescence and PA contrast agent due to its strong optical absorbance in the NIR region, and the intratumoral accumulation of NPs throughout the treatment could be monitored by the FI and PAI dual-modal imaging. In summary, this spatiotemporal controllable and biosafe theragnostic system was capable of ablating melanoma tumors in a single dose by integrating chemotherapy, PDT, and PTT, ultimately providing a promising candidate for the clinical treatment of melanoma and other superficial skin tumors.

## Data Availability

All data generated or analysed during this study are included in this published article.

## Abbreviations

AM: Acetoxymethyl
B-MN: Blank MNs
CLSM: Confocal laser scanning microscope
DCFH-DA: 2′,7′-Dichlorofluorescin diacetate
DOX: Doxorubicin
DOX·HCl: Doxorubicin hydrochloride
DSPE-mPEG2000: 1,2-Distearoyl-*sn*-glycero-3-phosphoethanolamine-*N*-[methoxy(polyethylene glycol)-2000]
FI: Fluorescence imaging
HPLC: High-performance liquid chromatography
HSPC: Hydrogenated soybean phosphatidylcholine
ICG: Indocyanine green
LC: Loading contents
IC_50_: Half maximal inhibitory concentration
IRT: Inhibition rate of tumor growth
MN: Microneedle
MTT: 3-(4,5-Dimethylthiazol-2-yl)-2,5-diphenyltetrazolium bromide
NIR: Near-infrared
NPs: Nanoparticles
NTDDSs: Nanocarrier-based transdermal drug delivery systems
PA: Photoacoustic
PAI: Photoacoustic imaging
pB-DOX: Prodrug of doxorubicin
PDMS: Polydimethylsiloxane
PDT: Photodynamic therapy
PI: Propidium iodide
PTT: Photothermal therapy
PVP: Polyvinylpyrrolidone
ROS: Reactive oxygen species
SC: Stratum corneum
SD: Standard deviation
SDrats: Sprague–Dawley rats
